# Catchment-based sampling of river eDNA integrates terrestrial and aquatic biodiversity of alpine landscapes

**DOI:** 10.1007/s00442-023-05428-4

**Published:** 2023-08-09

**Authors:** Merin Reji Chacko, Florian Altermatt, Fabian Fopp, Antoine Guisan, Thomas Keggin, Arnaud Lyet, Pierre-Louis Rey, Eilísh Richards, Alice Valentini, Conor Waldock, Loïc Pellissier

**Affiliations:** 1 Unit of Land Change Science, Swiss Federal Research Institute WSL, Birmensdorf, Switzerland; 2 Department of Environmental Systems Science, Institute of Terrestrial Ecosystems, ETH Zürich, Zurich, Switzerland; 3 Department of Aquatic Ecology, Eawag, Swiss Federal Institute of Aquatic Science and Technology, Dübendorf, Switzerland; 4 Department of Evolutionary Biology and Environmental Studies, University of Zurich, Zurich, Switzerland; 5 Department of Ecology and Evolution, University of Lausanne, Geopolis, Lausanne, Switzerland; 6 World Wildlife Fund, Wildlife Conservation Team, Washington, DC USA; 7 Institute of Earth Surface Dynamics, University of Lausanne, Geopolis, Lausanne, Switzerland; 8 SPYGEN, Le Bourget-du-Lac, France

**Keywords:** Biomonitoring, Environmental DNA, Spatial ecology, Metabarcoding, Biodiversity assessment

## Abstract

Monitoring of terrestrial and aquatic species assemblages at large spatial scales based on environmental DNA (eDNA) has the potential to enable evidence-based environmental policymaking. The spatial coverage of eDNA-based studies varies substantially, and the ability of eDNA metabarcoding to capture regional biodiversity remains to be assessed; thus, questions about best practices in the sampling design of entire landscapes remain open. We tested the extent to which eDNA sampling can capture the diversity of a region with highly heterogeneous habitat patches across a wide elevation gradient for five days through multiple hydrological catchments of the Swiss Alps. Using peristaltic pumps, we filtered 60 L of water at five sites per catchment for a total volume of 1800 L. Using an eDNA metabarcoding approach focusing on vertebrates and plants, we detected 86 vertebrate taxa spanning 41 families and 263 plant taxa spanning 79 families across ten catchments. For mammals, fishes, amphibians and plants, the detected taxa covered some of the most common species in the region according to long-term records while including a few more rare taxa. We found marked turnover among samples from distinct elevational classes indicating that the biological signal in alpine rivers remains relatively localised and is not aggregated downstream. Accordingly, species compositions differed between catchments and correlated with catchment-level forest and grassland cover. Biomonitoring schemes based on capturing eDNA across rivers within biologically integrated catchments may pave the way toward a spatially comprehensive estimation of biodiversity.

**Supplementary Information:**

The online version contains supplementary material available at 10.1007/s00442-023-05428-4.

## Introduction

With their complex and wide-ranging environmental conditions, mountainous regions host extraordinarily high biodiversity with high spatial turnover (Guisan et al. 2019; Kerr and Packer 1997; Körner 2004) while facing increasing anthropogenic pressures such as land-use intensification (Rounsevell et al. 2006). Large-scale biodiversity mapping tools are essential to control pressures on terrestrial landscapes and inform environmental policy decisions that can effectively enable sustainable land use (Cardinale et al. 2012; Isbell et al. 2017). However, though traditional site-based sampling methods are widespread and established, using these methods to sample biodiversity at larger spatial scales over regular intervals can become costly, time-intensive, and invasive. A less costly, non-invasive tool for rapid biodiversity assessment is environmental DNA (eDNA) metabarcoding. eDNA-based methods use DNA fragments that organisms have shed through faeces, skin cells, or organelles, captured through water or soil filtering (Ficetola et al. 2008). An eDNA approach could allow a more efficient assessment of biodiversity change and facilitate the survey of organisms at broad spatial and taxonomic scales. It also can potentially target the species assemblage of a region—such as a river catchment—for many groups (Altermatt et al. 2020) over regular periods (Deiner et al. 2017; Lawson Handley 2015). Hence, designing more efficient methods for integrative sampling of complex mountain terrains could provide a novel, efficient method to monitor biodiversity.

eDNA metabarcoding of riverine samples can be used to retrieve information on aquatic, semi-aquatic and terrestrial species in the wider landscape (Broadhurst et al. 2021; Deiner et al. 2016; Mizumoto et al. 2022; Ushio et al. 2017). Rivers can therefore function as “conveyor belts” of biological information stored in eDNA (Deiner et al. 2016; Sales et al. 2020; Villacorta-Rath et al. 2021), leading to an aggregated biodiversity signal downstream that captures the wider landscape biodiversity, i.e. biological integration. Because rivers exist in isolated hydrological catchments, such biodiversity signals should be broadly representative of wide-scale spatial units, as demonstrated in aquatic invertebrates, amphibians and other vertebrates (Deiner et al. 2016; Mizumoto et al. 2022; Villacorta-Rath et al. 2021). Nonetheless, eDNA in rivers has been predicted to show non-uniform concentration patterns along river networks due to hydrological conditions, and optimal site selection may depend on the taxonomic group (Carraro et al. 2021). Before eDNA-based methods are widely implemented into large biomonitoring programs, it is crucial to determine what portion of taxa occurring in a region (e.g., a catchment) an eDNA sample can represent.

An appropriate sampling design maximises the capture of the regional species pool while minimising the number of sites that require sampling (Altermatt et al. 2023). However, the number of spatial replicates required to get an accurate impression of a region can vary by site (Stauffer et al. 2021), as well as the taxon’s spatial abundance (Erickson et al. 2019) and distribution (Carraro et al. 2021). Assessing the whole biodiversity of an alpine river catchment requires knowledge of whether the eDNA of the species in habitats from higher elevations are integrated downstream, but this might depend on hydrological conditions (Altermatt et al. 2023). If this were the case, then sampling at the low elevation would provide an accurate enough representation of the whole catchment. If the signal remains primarily localised, then the information from higher elevation habitats and their species would not be reflected within the low elevation sample, and more sites upstream would be necessary. Understanding how information about entire species assemblages is integrated downstream is critical for an accurate assessment of catchment-scale biodiversity.

The use of eDNA in the context of biodiversity assessment has already been applied to vertebrates and invertebrates for various ecosystem quality assessments (Keck et al. 2022). Contrastingly, it is less known whether terrestrial plants can also be captured using eDNA metabarcoding of river water (Mächler et al. 2019; Seymour et al. 2020). Yet, plant composition can be very informative for evaluating the ecological quality of a site (Spyreas 2019). Plant inventories play an important role in evaluating ecosystem functionality and recovery (Abe et al. 2021; Bachand et al. 2014), such as food resources for species at higher trophic levels (Felton et al. 2010). To better evaluate ecosystem health, plant assemblages must be sampled, and not just the animals depending on them. We know from eDNA metabarcoding in soil samples that it can simultaneously identify seeds (active and dormant), pollens, and detritus of species, thus providing a quick and comprehensive overview of terrestrial plant composition and diversity (Fahner et al. 2016; Yoccoz et al. 2012). Moreover, river and lake samples have already been able to accurately capture the composition of aquatic vascular plant assemblages and identify invasive species (Coghlan et al. 2021). Rather than sampling in different substrates such as water and soil whilst increasing sampling time and effort, using multiple primers for animals and plants within the same river sample could be a time and cost-effective method of monitoring the many aspects of biodiversity across spatially heterogeneous river catchments.

In this study, we investigated mountain rivers within ten hydrological catchments in the Western Swiss Alps to determine how eDNA sampling can capture the diversity of a region and within which spatial neighbourhood. We targeted mainly terrestrial clades by sequencing two widely different groups of organisms, spermatophytes and vertebrates, using two different primer sets. We sampled different river locations across a wide elevational gradient within each catchment to investigate whether the signal is localised or integrated downstream in the catchment. We compared the species recovered to the best existing estimate of the regional species pool. We aimed to answer the following questions:

1. How many sample replicates are required per catchment to reach saturation when assessing the regional diversity (γ-diversity)?
2. How well do samples from rivers at lower elevations integrate biological signals from species present at higher elevations?
3. Can the distinct assemblages present in each catchment and elevation be identified using an eDNA approach?

## Materials and methods

### Sampling design and water filtration

**Fig. 1:**
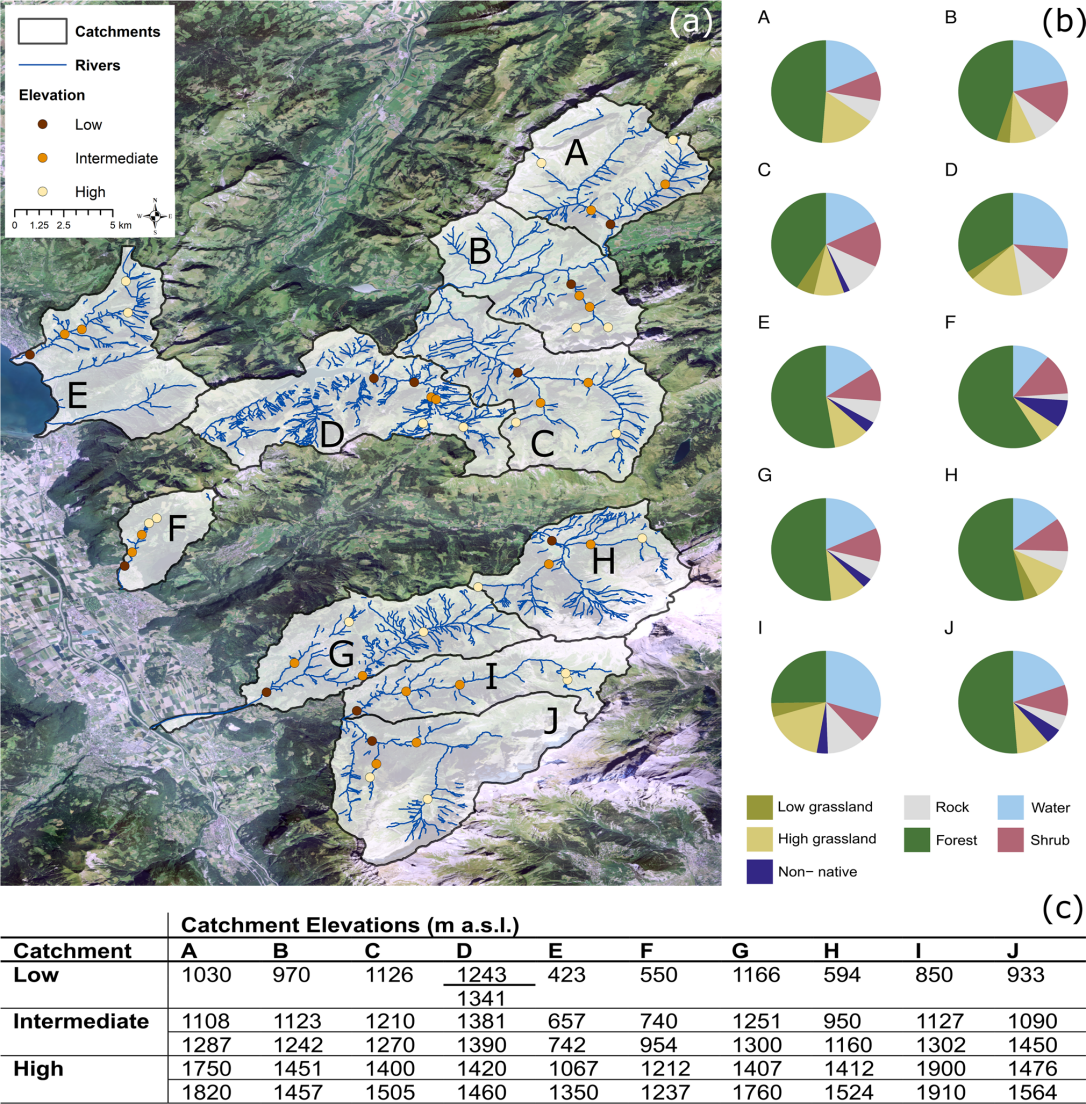
Study Area and plant assemblage composition. A map (**a**) of the study area (FOT swisstopo 2021) showing the visited sites overlaid on polygons of the rivers within the catchments (FOEN 2010). Pie charts (**b**) represent the proportion of plants detected in the eDNA samples by land cover type, based on a modified *Flora Indicativa* plant assignment (see Supplementary Information 3). The elevations of the sites visited are presented in the table (**c**), and the catchment IDs correspond to the map and pie charts

We selected ten catchments [based on Topographical catchment areas of Swiss water bodies 40 km^2^ (FOEN 2010)] of the Northern Alps biogeographic region in the canton of Vaud, Switzerland, a region where plant and animal biodiversity have historically been well-documented (Dubuis et al. 2013; Pellissier et al. 2013). Moreover, its comparatively high heterogeneity of vegetation types and various land uses enables the potential to distinguish each catchment's plant assemblage (Randin et al. 2009). From 22-Jun-2020 to 26-Jun-2020, we sampled five sites comprising one low, two intermediate and two high-elevation sites per catchment (Fig. 1a). We visited two catchments per day—one in the morning and one in the afternoon—for a total of ten catchments. All samples per catchment were collected within a maximum four-hour period by three groups of samplers. The intermediate and high-elevation sites were situated along two tributaries leading into the low-elevation site of the river. We used three filters for each relative elevation class and filtered 60 L per relative elevation class. We sampled 30 L per tributary for a combined volume of 60 L at the intermediate and high-elevation sites. In total, 180 L were sampled in total per catchment. A filtration device composed of either the Athena^®^ peristaltic pump (Proactive Environmental Products LLC; 1 L/min nominal flow) or the Subspace^®^ underwater peristaltic pump (Subspace Pictures; 1 L/min nominal flow), combined with a VigiDNA^®^ 0.2 µM cross-flow filtration capsule (VigiDNA, SPYGEN) was used in order to filter a large water volume. We used a finer mesh than the recommended VigiDNA^®^ 0.45 µM cross-flow filtration capsule to maximise the capture of biological material since mountain water does not transport high quantities of sediments. For each filtration capsule, we used disposable sterile tubing. At the end of each filtration, we emptied the water inside the capsules, replaced it with 80 ml of CL1 conservation buffer (SPYGEN), and stored it at room temperature. We followed a strict contamination control protocol in both field and laboratory stages (Goldberg et al. 2016; Valentini et al. 2016). Each water sample was processed using disposable gloves and single-use filtration equipment. We used two primer sets targeting vertebrates (Vert01, forward: − TTAGATACCCCACTATGC, reverse: − TAGAACAGGCTCCTCTAG, mean marker length: 97 bp) and spermatophytes (g-h/Sper01, forward: − GGGCAATCCTGAGCCAA, reverse: CCATTGAGTCTCTGCACCTATC, mean marker length: 48 bp) (Taberlet et al. 2018). Though both primers are relatively broad with low species-level resolution, we selected them as the goal was to minimise cost and effort and maximise the identification of a broad range of taxa which can represent the species assemblages of the region. We used existing protocols (Polanco Fernández et al. 2021; Valentini et al. 2016) to perform eDNA extraction, PCR amplification, purification and library preparation, as well as bioinformatic analyses (See Supplementary Information 1 for more detail).

### Species accumulation curves and comparison with conventional observational surveys

Using the outputs of the bioinformatic analyses, we evaluated whether the spermatophyte and vertebrate taxa at all resolutions recovered with the two primers matched the species recorded by national catchment-level occurrence datasets. These surveys represent an archive of long-term (multiple decades, up to centennial for some taxa) ongoing monitoring efforts and thus were not performed at the same time as the eDNA-based sampling. Those represent the best available information about these taxa at the catchment level. As we aimed to determine the extent of existing knowledge of biodiversity in the region, which could be captured by a short field excursion using eDNA sampling, we excluded all taxa not represented in the occurrence datasets for each catchment. These excluded taxa could be false assignments, horticultural or agricultural species, or potentially introduced species.

We additionally corrected for taxonomic redundancy following (Marques et al. 2020) by only including higher order taxa if there were no species already belonging to the rank, as this could have resulted in nested taxa and over-inflation of taxon richness. Unlike (Marques et al. 2020), our reference databases for some groups were significantly incomplete, and thus higher-order taxa may represent species present and captured in the region but absent from the reference database. Thus, we modified their approach to only exclude taxa if all species nested within the taxa and known to occur in the region (Supplementary Information 2) were accounted for by eDNA at the regional scale (all ten catchments).

We calculated regional species richness as the sum of all species present in the ten catchments and classified them according to their abundances to obtain the regional richness of common species (Supplementary Information 1). We additionally classified all identified taxa as common or rare (Supplementary Information 1). For amphibians, birds, fish, mammals, and spermatophytes, we used taxon richness as a proxy for regional species richness and compared this with the expected total regional species richness according to the existing records. Moreover, we compared common taxon richness to the expected common regional species richness. We used taxon richness as the primers do not have a high species-level resolution, and some species were not present in the reference database used for the bioinformatic analyses. We define taxon richness as the richness of all taxa in all ranks, including and under the taxonomic group of interest (amphibians, birds, fish, mammals, and spermatophytes). We produced taxon richness accumulation curves for both all and only common species across filtration replicates for the region (the sum of all ten catchments) using the R package *vegan* and its *specaccum* function (Oksanen et al. 2020). We generated 1000 accumulation curves using the ‘random’ method to fit models that describe the relationship between taxon richness and replicates (number of filters required). We fitted fourteen models to each saturation experiment and ranked them by AIC score. We then generated multimodel mean averages, which, along with the *sars_average* function (R package *sars* version 1.3-7), ﻿were used for extrapolation (Matthews et al. 2019). Then the *sar_pred* function was used to extrapolate taxon richness for up to 50 filtration replicates. We calculated the number of filtration replicates required to reach regional species richness (total and common), up to fifty replicates, as further extrapolation would incur increased uncertainty. In order to test the sensitivity of this analysis to the inclusion of the taxa which had been classified as redundant, we repeated this analysis without excluding the taxa.

### Partition of beta diversity between elevations

To quantify the dissimilarity in species composition between elevations in catchments, we calculated beta diversity and partitioned it into its turnover and nestedness components. We utilise the definition of beta diversity as the sum of species replacement between the sites (turnover) and the site-to-site species loss (nestedness) (Baselga 2010). High turnover from upstream to downstream would indicate that biological information remained highly localised, as the species composition would change from site to site. Conversely, high nestedness, where the upstream species composition is a subset of the downstream species composition, would indicate that biological information is integrated downstream in the low-elevation site. We calculated the Jaccard dissimilarity index to partition beta-diversity into these two components using the *betapart.core* function in the R package *betapart* version 1.6 (Baselga and Orme 2012). We calculated nestedness and turnover between the low and intermediate elevations and the intermediate and high elevations to determine the extent to which eDNA conveyed biological information differently between the two sections of the river system. We tested whether the differences in nestedness and turnover between elevations were higher between low and intermediate versus intermediate and high classes and also between taxonomic groups using Wilcoxon Rank Signed Tests.

### Assemblage composition across catchments

We conducted a principal coordinates analysis (PCoA) to determine whether clear compositional differences between the catchments and elevations can be evidenced in vertebrate and spermatophyte assemblages. We used the *dudi.pco* function in the *ade4* package (version 1.7-20) in R (Dray and Dufour 2007), with the distances calculated as a Euclidean distance dissimilarity matrix based on the Jaccard index. We computed the explained deviance. We then carried out a distance-based redundancy analysis (dbRDA) with the eigenvalues obtained in the PCoA, using the *capscale* function in the R package *vegan* version 2.6-4. We tested the significance of the canonical axes of the dbRDA using the Monte Carlo permutation test (*anova.cca* function in R package *vegan*), using relative elevation and catchment as constraints. In order to determine the extent of the spatial neighbourhood covered by the samples, we also used the fraction of land cover type per catchment and per circular buffers around the sites with radii of 250 m, 500 m, and 1000 m. The relative land cover variables were computed using the remotely sensed land use map *Arealstatistik nach Nomenklatur 2004, Erhebungen 1979–1985, 1992–1997, 2004–2009, 2013–2018* (henceforth *Arealstatistik)* at 100-m resolution for the 2013–2018 samples (Bundesamt für Statistik (BFS), 2021). We reclassified the 72 classes according to *Nomenklatur 2004* into seven land cover types (forest, high elevation grassland, low elevation grassland, rock, shrubland, urban and water bodies (Supplementary Information 3) and computed the fraction of each land cover per catchment. We created circular buffers around the sites with radii of 250 m, 500 m, and 1000 m and computed the fraction of each land cover per site using the *st_buffer* function in the R package *sf* version 1.0-9 (Pebesma 2018)*.* We correlated these compositions to the relative proportion of land cover types within each catchment and within the circular buffers according to the remote sensing data using the Kendall Tau-B statistic.

Moreover, plant genera present in the samples can be assigned to land cover types according to a modified *Flora Indicativa* habitat classification system (Landolt et al. 2010) (Supplementary Information 3), which we related to catchment properties. In Switzerland, an established method is the usage of *Flora Indicativa: Ecological indicator values and biological attributes of the Flora of Switzerland and the Alps* (Landolt et al. 2010)*.* We used the plant community classifications of *Flora Indicativa* and focused on indicator genera (those present in 5 or fewer communities) to characterise the relative habitat compositions of the ten catchments. We used the genus level because the taxonomic resolution of the *Sper01* primer was poor at the species level. Moreover, non-native spermatophyte genera were assigned a new class of "non-native" outside the *Flora Indicativa* classification. Then, we calculated relative fractions of each *Flora Indicativa* habitat per catchment as the sum of plants assigned to each habitat category. We investigated whether the number of spermatophyte genera assigned to these categories correlated with the proportions obtained from *Arealstatistik* (at the catchment, 250, 500 and 1 000 m radii buffer scales) using the Kendall Tau-B Rank correlation coefficient for alpine grassland, lowland grassland, forest, and rock fractions, shrub, water. Moreover, we investigated whether the proportion of urban cover correlated with non-native spermatophyte genera present in each catchment (Supplementary Information 3).

All statistical analyses were performed in R 3.6.2. (R Development Core Team 2011).

## Results

We detected 349 unique taxa assigned to vertebrate (86) and spermatophyte (263) taxa (see Supplementary Information 2 for a full list of taxa) across the ten catchments, which we defined together as our study region. Vertebrate taxa were distributed in 3 amphibian (5 taxa), 18 avian (37), 6 fish (14), 13 mammalian (29) and 1 reptilian (1) families. Plant taxa were distributed in 68 angiosperm (243), 3 conifer (8), 1 fern (2) and 7 moss (10) families. However, since the *Sper01* primer is not optimal for amplifying non-seed-bearing plant species, mosses and ferns were excluded from further analyses.

### Regional diversity assessment

**Fig. 2:**
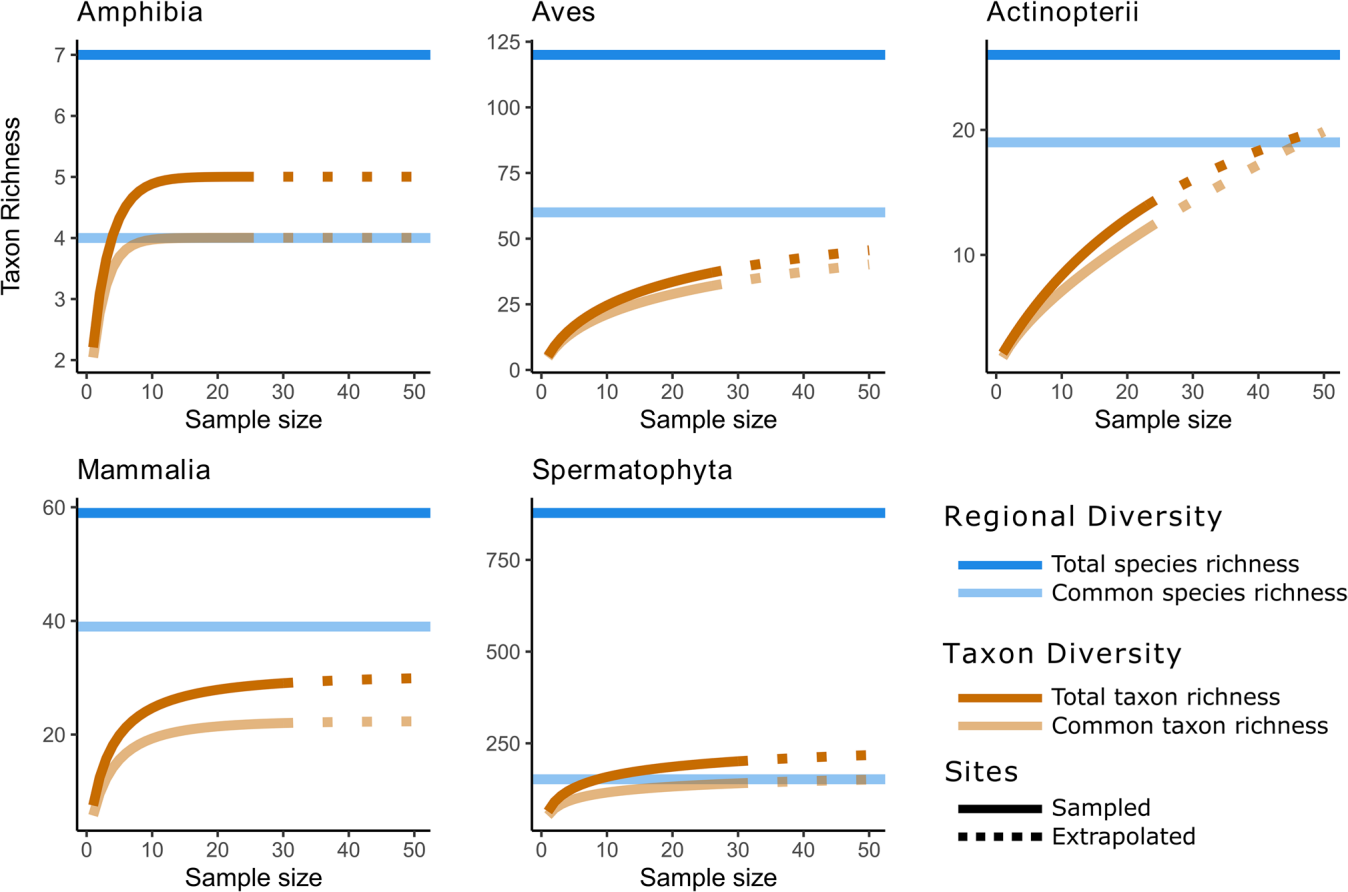
Species accumulation curves across filter replicates for amphibians, birds, fishes, mammals, and seed-bearing plants. The darker blue line represents total regional species richness, and the lighter blue line represents common regional species richness. The solid curves represent multimodel mean averages for the 30 replicates sampled, while the dashed curves represent extrapolated values for a further 20 replicates. The darker shades represent all found taxa, while the lighter shades represent common taxa

In our assessment of regional diversity captured by eDNA sampling, we found that common species' regional richness was reproduced by amphibians’ common taxon richness (4/4 common species within thirty filters). Within thirty filters, the regional richness of common spermatophyte species was nearly reproduced (141 taxa/152 common species). This was not the case for mammals (22 taxa/39 common species), birds (32 taxa/60 common species) or fishes (12 taxa/19 common species) within thirty filters. At the regional scale, the taxon richness accumulation curves reached a plateau for mammals, amphibians and spermatophytes. They did not flatten for fishes and birds within the 30 filters (Fig. 2), but some reached an asymptote when extrapolating to 50 filters. For birds, neither regional nor common regional species richness is reached by 50 replicates. Of the 86 vertebrate taxa, 71 taxa were regionally common. Additionally, 50/86 taxa were identified to the species level, of which 40 were regionally common (80%). For amphibians, eDNA successfully detected five out of seven species known to the area, including all four species common to the area and the rarer *Salamandra salamandra.* The two species not detected were the rare and potentially close-to-extinct *Bombina variegata,* as well as *Pelophylax esculentus.* For birds, it is expected that the success rate would be lower than for groups that are more closely associated with water, which is on par with the finding of only 37 taxa, 13 at the species level out of 120 bird species expected to occur across these ten catchments*.* Regionally rare mammals, such as *Microtus agrestis, Microtus subterraneus, Neomys fodiens,* and *Sorex araneus,* were also detected at the species level. For fish, seven detections were at the species level. We detected 57 spermatophyte species with known distributions in these catchments, of which 33 could be classified as common or rare using abundance data. Of these 33 species, 20 were regionally rare (61%). 57% of identified genera (48/84) were common to the region. Of the 263 spermatophyte taxa, 201 could be classified as common or rare, of which 141 taxa (70%) were common to the region. Though rare taxa were indeed detected, a larger proportion of the eDNA signal represents common taxa in the region.

### Species compositional dissimilarity between elevational classes

**Fig. 3:**
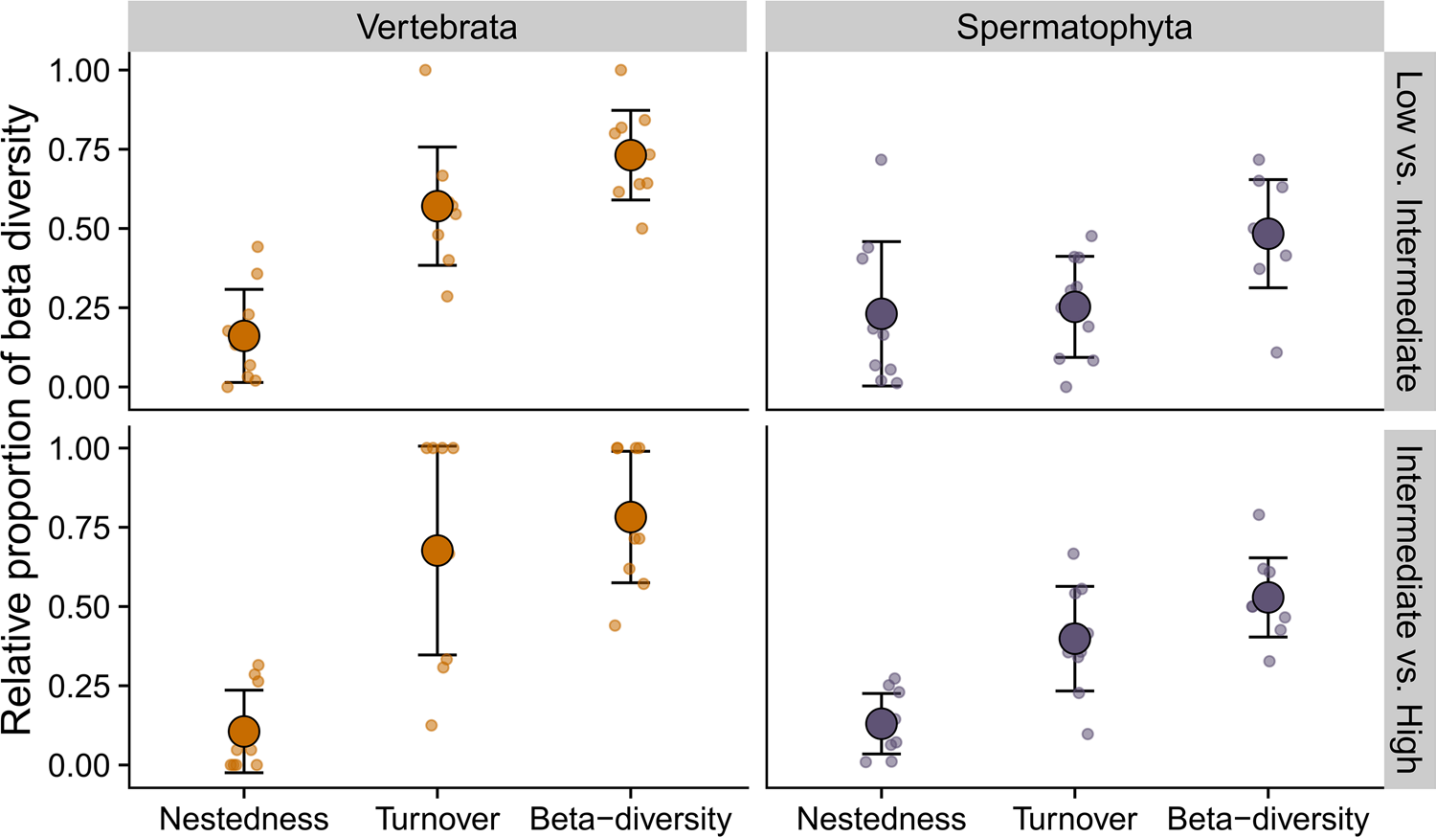
Species compositional dissimilarity (β-diversity) between low and intermediate sites and intermediate and high sites and their partitioning into nestedness and turnover. The large orange (vertebrates) and purple (seed-bearing plants) circles represent the mean nestedness and turnover of the ten catchments, and error bars represent the standard deviation. The small translucent circles represent the individual values per catchment

The composition of vertebrate species was highly dissimilar between both the low vs intermediate elevation (mean *β-*div = 0.73 ± 0.14) and intermediate vs high elevation pairs (mean *β*-div = 0.78 ± 0.21), where a value of 1 indicates complete dissimilarity with no species in common between elevational classes (Fig. 3). The composition of spermatophyte species was dissimilar between both the low vs intermediate (mean *β*-div = 0.48 ± 0.17) and intermediate vs high pairs (mean *β*-div = 0.53 ± 0.12). When comparing the β-diversity of vertebrates and spermatophytes (Fig. 3), vertebrates had higher compositional dissimilarities between the low and intermediate elevation sites (Wilcoxon Signed Rank Test, *n* = 20, *Z* = − 2.89, *p* < 0.01), and between intermediate and high elevation sites (Wilcoxon Signed Rank Test, *n* = 20, *Z* = − 3.09, *p* < 0.01).

For vertebrates, species compositional differences between the elevational classes were primarily due to species turnover for both low vs intermediate (Wilcoxon Signed Rank Test, *n* = 20, *Z* = − 2.58, *p* < 0.01, mean turnover = 0.57 ± 0.19) as well as intermediate vs high comparisons (Wilcoxon Signed Rank Test, *n* = 20, *Z* = − 2.46, *p* = 0.01, mean turnover = 0.68 ± 0.33). Nestedness contributed less in both cases as well, with mean values being 0.16 ± 0.15 (low vs intermediate) and 0.10 ± 0.13 (intermediate vs high). Spermatophytes contrasted these patterns, whereby species compositional differences between the low and intermediate elevation classes were almost equally due to nestedness (mean = 0.23 ± 0.22) and turnover (mean = 0.25 ± 0.15) with no significant difference between the two (Wilcoxon Signed Rank Test, *n* = 20, *Z* = -0.39, *p* = 0.69). In the intermediate vs high elevation comparison, the turnover component (Wilcoxon Signed Rank Test, *n* = 20, *Z* = − 2.46, *p* = 0.01, mean turnover = 0.39 ± 0.16) contributed significantly more to the β-diversity than nestedness (mean nestedness = 0.13 ± 0.10) (Fig. 3).

### Detection of distinct vertebrate and spermatophyte assemblages

**Fig. 4:**
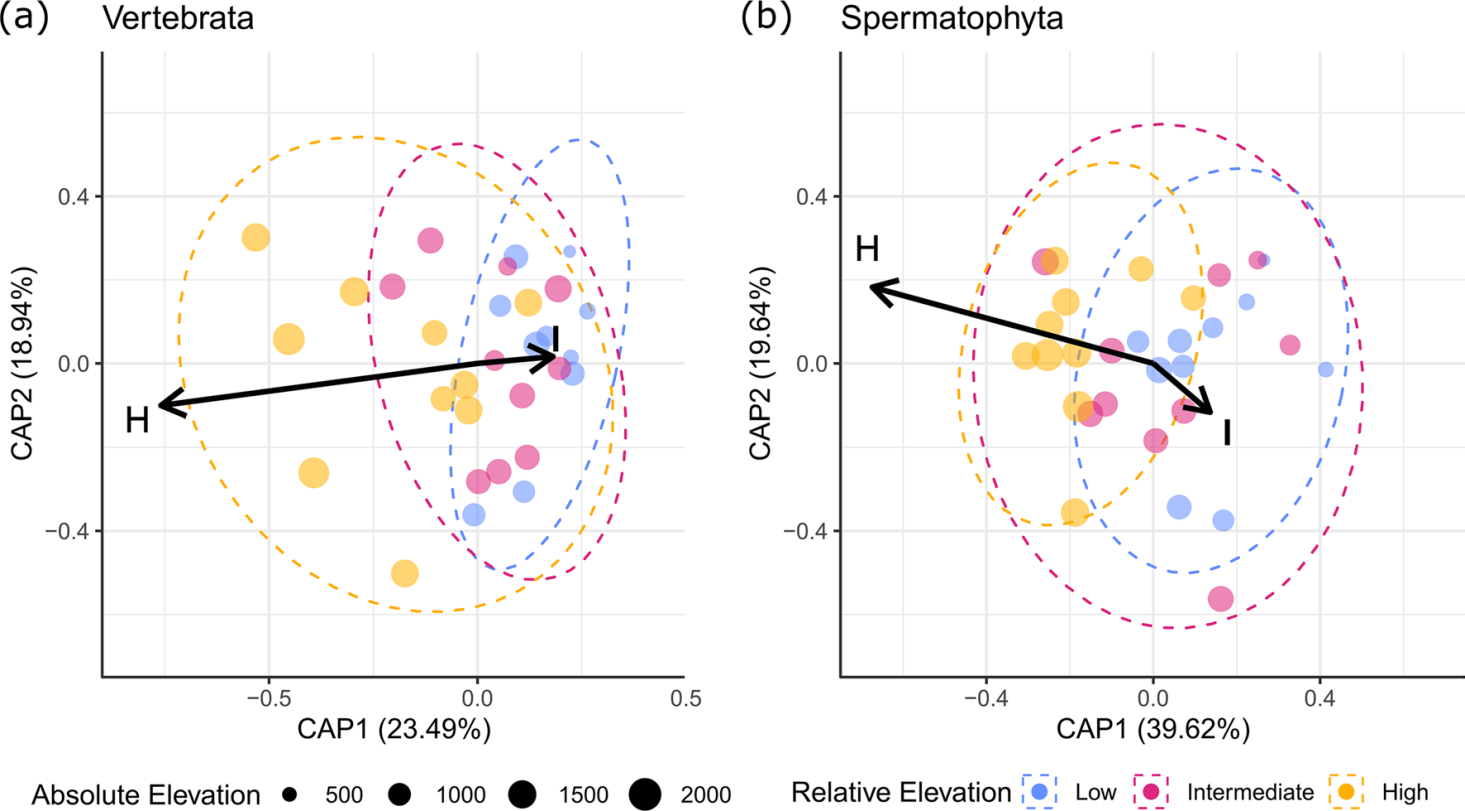
Distance-based redundancy analysis (Canonical Component Analysis) of site species compositional dissimilarity showing the changes with relative elevation (black arrows) for (**a**) vertebrates and (**b**) spermatophytes. The relative elevation classes were low, intermediate (I), and high (H). Distances were calculated using the Jaccard dissimilarity index. Points represent sites, and the ellipses in dashed lines represent the ordiellipses (standard error with 95% confidence interval)

Both vertebrate and spermatophyte assemblages were significantly constrained by catchment and elevation (Table 1), indicating that the assemblages were compositionally different between catchments and elevations. When comparing the dissimilarities between vertebrate assemblages across all filters in the region to catchment land cover characteristics, we found that the first two axes explained 42.43% of the variation (Fig. 4a). Relative elevation, proportion of forest, shrubland, low grassland and high grassland per catchment were significant constraints on the dissimilarity of vertebrate species compositions (Table 2). The first two axes explained 59.26% of spermatophyte assemblages (Fig. 4b). Relative elevation, proportions of forest, urban and high grassland cover per catchment were significant constraints (Table 2). Overall, the canonical axes were highly significant for both vertebrates (Monte Carlo permutation test: *F*_(8,21)_ = 1.50, *p* < 0.001) and spermatophytes (Monte Carlo permutation test: *F*_(8,21)_ = 2.04, *p* < 0.001). When comparing the dissimilarities between vertebrate assemblages across all filters in the region to land cover characteristics in the nearer neighbourhood of 250, 500 and 1000 m buffers, no constraints were significant (Supplementary Information 3). However, for spermatophytes, fractional water cover was a significant constraint at 250 m, while absolute elevation was significant at 250 and 1000 m and showed a trend at the 500 m scale (Supplementary Information 3).

**Table 1:** Monte Carlo permutation test results for dbRDA on factors catchment and relative elevation

Group	Constraint	Df	Sum of Sqs	F	Pr (> F)
Vertebrates	Catchment	9	3.701	1.380	0.001
	Relative elevation	2	0.904	1.517	0.003
	Residual	18	5.365		
Spermatophytes	Catchment	9	2.153	1.760	0.001
	Relative elevation	2	0.659	2.423	0.002
	Residual	18	2.446		

We assessed the marginal effects of relative elevation and catchment on the 30 vertebrate and spermatophyte assemblages using the Jaccard dissimilarity index

**Table 2:** Monte Carlo permutation test results for dbRDA on catchment-level land cover proportions and elevation

Group	Predictor	Df	Sum of sqs	F	Pr (> F)
Vertebrates	Forest	1	0.520	1.722	0.008
	High grassland	1	0.542	1.795	0.007
	Low grassland	1	0.463	1.532	0.040
	Shrubland	1	0.464	1.536	0.030
	Urban	1	0.387	1.282	0.121
	Water	1	0.432	1.429	0.068
	Relative elevation	2	0.904	1.496	0.004
	Residual	21	6.344		
Spermatophytes	Shrubland	1	0.321	2.282	0.011
	High grassland	1	0.278	1.974	0.018
	Low grassland	1	0.191	1.356	0.120
	Shrubland	1	0.198	1.409	0.103
	Urban	1	0.263	1.870	0.023
	Water	1	0.162	1.149	0.250
	Relative elevation	2	0.659	2.338	0.003
	Residual	21	2.958		

We assessed the marginal effects of the relative elevation and fractions of land cover (forest, high grassland, low grassland, urban, shrub, water) on the vertebrate and spermatophyte assemblages using the Jaccard dissimilarity index. As the fraction of rock cover was highly correlated to the fraction of forest, we removed it from this analysis

The relative composition of the broad *Flora Indicativa* land cover types assigned from the captured spermatophyte genera differed across the ten catchments (Fig. 1b). At the catchment level, the relative composition of land cover types did not significantly correlate to the relative compositions of land cover types captured by the eDNA samples (Supplementary Information 3). At 250 m and 500 m, the fraction of high grassland cover was significantly correlated to the relative composition of land cover types captured by the eDNA samples (Supplementary Information 3).

In a parallel analysis that did not correct for taxonomic redundancy, all results were comparable to those that excluded them. One notable exception is that the inclusion of these higher taxa resulted in the common regional species richness of mammals being captured in the regional diversity assessment analysis (Supplementary Information 3).

## Discussion

Focusing on specific taxonomic groups, we demonstrate that catchment-level terrestrial biodiversity can be reliably estimated through samples of eDNA from riverine water. Relying on five days of sampling across fifty locations, we show that taxon richness for amphibians and spermatophytes was comparable to regional common species richness within thirty replicates. Contrary to our expectations and the idea that biological information can be conveyed via rivers at large distances (Deiner et al. 2016; Deiner and Altermatt 2014), the species composition of higher elevation sites was not significantly nested within the lower elevation sites, except between the low and intermediate elevations for spermatophytes. We identify the unique land cover configurations of the ten catchments and show that the species composition was significantly correlated to the proportional cover of some land cover types. Our work supports that sampling of riverine eDNA offers an unrivalled rapid assessment of terrestrial biodiversity toward landscape-scale assessments. Though intensification of sampling volume, time of filtration or number of samples per catchment is necessary to estimate catchment-level biodiversity better (Altermatt et al. 2023), our results demonstrate that regional diversity could be estimated using eDNA with relatively few samples during short field visits.

Our results support the ability of eDNA metabarcoding to recover amphibians well, as shown in other monitoring investigations (Manzer et al. 2020). Only half of the other groups of vertebrates (i.e. mammals, birds, and fishes) potentially occurring in the areas were detected. However, the list of potential species in each catchment represents the accumulated knowledge of decades of observation. Hence, the detection of nearly half the common regional taxa within a 5-day sampling period (mammals: 56%, birds: 53%, fishes: 63%) is already a promising result for catchment-level biodiversity monitoring using eDNA from river water. While the recovery was high for amphibians, in the case of mammals and birds, the inability to detect some species may be due to their behaviour with limited interaction with water (De Souza et al. 2016) or because they may not be present anymore in the study area following anthropogenic activities (Tingley and Beissinger 2009). With regards to fish taxa, while we considered the whole catchments to establish the potential fish checklist, the sampling was more intensively focused on the upper stretch of the catchment, which is suitable to only a small subset of these species (Askeyev et al. 2017). Thus, we hypothesize that an additional lower elevation site in the true lowlands would have improved the rate of fish taxa recovery. We detected one reptilian taxon: *Zootoca vivipara,* and no higher-level taxa. We surmise that reptiles may have a lower shedding rate than other organisms like fish and amphibians and have more limited contact with water, so they are not an ideal group for detecting by river water (Nordstrom et al. 2022). We additionally recovered 93% of the common spermatophyte taxa in the region. The highly heterogeneous topography of the alpine catchments has led to an extraordinary diversity of plants in the area (Rahbek et al. 2019); this total diversity could not be captured by this present sampling approach. Additionally, unlike vertebrates, most plant species employ less direct contact with river water. Therefore, they may be better suited for sampling using soil (Yoccoz et al. 2012) or following periods of high concentrations of pollen in the air, which have been documented to sink into the water surface with time (Pansu et al. 2015). At the regional level, a larger number of samples may be required to inventory the total fauna and flora of the region, including more rare species.

While it has been established that rivers can transport eDNA downstream (Deiner et al. 2016; Deiner and Altermatt 2014; Pont et al. 2018), challenges remain in assigning the location and spatial scale of the biological signal. Our results show the species composition of higher elevation sites was not substantially nested within that of the lower elevation sites, in contrast with a previous study on mammals on a catchment in British Columbia (Lyet et al. 2021). Lyet et al. (2021) demonstrated that the regional terrestrial mammal biodiversity signal was integrated with relatively few samples in larger, faster and more turbulent streams with high sediment transport potential so that sampling a large volume downstream of the catchment was sufficient to obtain most of the diversity. The transport of eDNA downstream depends on river size, depth, and velocity, where larger, deeper and faster rivers convey eDNA further downstream to provide a more spatially integrated measure of biodiversity (Pont et al. 2018). In the present system, the alpine tributaries in this system—especially those in the high-elevation sites—are relatively small and shallow, potentially leading to a decreased rate in the transport of sediments and eDNA downstream. Downstream transport of eDNA over longer distances may be reliant on the attachment of eDNA to sediment particles (Behnke et al. 2023). Thus, the downstream transport of capturable eDNA becomes limited in high-alpine systems by the lower ability of the rivers to transport sediment particles. Moreover, higher elevation sites may house more animal and plant species (Körner 2004; Myers et al. 2000; Rahbek 1995) than the intermediate and low elevation sites. In such alpine streams, exclusive downstream sampling may thus be a less reliable approach to estimate whole catchment diversity.

Combining eDNA with ecological indicator species information can allow for ecosystem health assessments in managed landscapes in a repeatable, quantitative way (Siddig et al. 2016; Spyreas 2019). Therefore, combining eDNA metabarcoding with indicator information could generate a landscape-scale assessment of ecosystem composition and health and their changes over time (Blattner et al. 2021; Siddig et al. 2016). Here, we found that the fraction of land cover types at the catchment level was not significantly correlated with the spermatophyte species assigned to them using the *Flora Indicativa* method. We explain this poor association by the eDNA from high alpine sites might not be transported very far by water, creating a local rather than catchment-wide signal. Using more local analyses, we found that the proportion of high grassland species around each sampling site (buffers of 250 and 500 m radii) in eDNA was significantly correlated with the proportion of high-elevation grassland cover. As there was no animal equivalent for *Flora Indicativa,* a parallel analysis could not be performed for the vertebrates. Irrespective of the *Flora Indicativa* classes, the dbRDA demonstrated that species composition in eDNA generally reflects land cover fractions across catchments, especially forest and high grasslands for both vertebrates and spermatophytes. In the future, combining indicator values, such as habitat commonness and invasions (Djurhuus et al. 2020; Godefroid and Koedam 2003), for animal and plant species, with eDNA data could offer an efficient tool for monitoring ecosystems.

Considering the relative localisation of eDNA between the sites in this present study, repeated monitoring efforts along alpine mountain streams can establish a time series that elucidates mountain species shifts to higher elevations as a response to warming. Alternatively, by focusing on indicator species representing crucial plant communities (Bunce and Freeth 2022), it may be possible to identify catchments of conservation importance and inform land-use change strategies to mitigate their loss (Carignan and Villard 2002). Such an approach would not require massive sampling efforts to capture all species in a catchment and instead utilise a more judicious, cost-effective approach. A similar use of targeted primers designed for known potentially invasive species can also serve as an early warning signal for conservationists and drive prompt actions (Blackman et al. 2018). Though this present study focused on how well our method compared to existing species knowledge, non-native species had also been identified (Supplementary Information 2), such as the *Buddleja* genus, which may point towards the presence of *Buddleja davidii,* an invasive neophyte. eDNA-based monitoring approaches can create a cost-effective opportunity to sample a region quickly and repeatedly, which is especially difficult in less accessible regions such as alpine mountains, for the identification of native and non-native species, as well as entire communities (Lin et al. 2021) and enable policy-makers to make decisions based on the most recent snapshots of the region.

## Limitations

eDNA-based sampling is a potentially powerful tool for capturing regional diversity; however, the study design must be improved to reach a more comprehensive regional estimate. In this present study, common regional species richness was well-captured for most groups, but total regional species richness was underestimated in some groups. Recent work highlights that both the volume of filtered water and the duration of filtration affect the probability of species detection (Lyet et al. 2021). Though we filtered comparable volumes of water to Lyet et al. (2021) (60 L vs 70–80 L, respectively), our duration was significantly shorter (1-h vs up to 6-h), which may have decreased the chance of events where an organism interacts with filtered water. Moreover, although the number of sites we used captured the common regional species richness relatively well, more intensive sampling would be required to estimate richness within each catchment.

A one-time sampling of eDNA at a site provides a snapshot of a moment in time (Yamamoto et al. 2016) without considering eDNA release, diffusion, and degradation patterns and how this is influenced by seasons and weather conditions. For example, it has been estimated that 92–99% of the recovered eukaryotic species information could be detected 4–6 km downstream, but only on rainy summer days (Yang et al. 2021). Species composition has also been shown to be directly affected by seasonality and temporal turnover, using both eDNA-based and traditional biomonitoring methods (Seymour et al. 2021). Therefore, further studies that consider climatic or hydrological variables would enable a clearer understanding of how terrestrial eDNA is released, transported, and degraded as it travels downstream in a river catchment.

Effective identification is especially challenging for plants as universal primers suitable for eDNA analysis with high-resolution levels do not exist. The ideal eDNA metabarcoding marker should be variable, standardised, phylogenetically informative, extremely robust and short. Unfortunately, such an ideal DNA marker does not exist, so when working with environmental samples, the primer robustness and shorter fragments should be favoured (Valentini et al. 2009), but this will result in a loss of taxonomic resolution. However, despite this disadvantage, the primers used in this study are highly conserved, and they guarantee a robust amplification system without amplification bias between the different spermatophyte species (Taberlet et al. 2007). One approach that improves taxonomic resolution is to complement the analysis with additional primer pairs specifically designed to amplify more discriminant genetic regions for families with poor resolution levels (Baamrane et al. 2012; De Barba et al. 2014). However, a targeted metagenomic approach with a focus on bioindicator (species with specific habitat associations that are useful for habitat classification) (Blattner et al. 2021; Kuzmina et al. 2018), or invasive (Shackleton et al. 2019) plant species, have been proven successful for some groups. With the broad primer, we detected *Pedicularis ascendens* in one high-elevation site. This is a regionally rare species and indicative of only one *Flora Indicativa* plant community: “6.5 subalpine-alpine lawns in the broader sense, including rocky lawns”. Consequently, using a more targeted marker may lead to more effective species-level identification of terrestrial indicator plants and a clearer understanding of plant communities in the catchment.

More targeted and efficient primer choices would also result in higher species-level identification of vertebrate target groups (Lyet et al. 2021; Sales et al. 2020; Shackleton et al. 2019). Although eDNA metabarcoding of rare species is rapidly becoming more common, insufficient specificity in the primer can reduce the identification accuracy, especially when evolutionarily related species are present in abundance (Wilcox et al. 2013). This study found no critically endangered or endangered species from the Swiss Red List of Species (FOEN 2021). In future studies, the use of multiple targeted vertebrate primers for amphibians, fish, mammals, and birds, along with family-specific plant primers, may increase the taxonomic resolution of the sample results and enable better detection of rare species.

eDNA-based identification of species is only as good as the level of completeness of the reference database. Accurate identification of species assemblages requires a reference database that covers the region's biodiversity well. This coverage varied substantially for the different taxonomic groups covered in this study. For example, the reference database was particularly good for amphibians (7/7 species, 100%), fish (25/26 species, 96%), mammals (49/58 species, 84%), and spermatophytes (689/878 species, 78%), while less ideal for birds (70/122 species, 57%). For example, the regionally common *Sorex alpinus* species could not be detected as it was not present in the reference database, though the *Sorex* genus was detected in multiple catchments. Thus, using a local reference database may allow for increasing the taxonomic resolution of the assigned eDNA sequences [e.g. (Taberlet et al. 2007)]). This would also minimise over-inflation of taxon richness due to taxonomic redundancy from higher order taxa, as they could be (a) completely removed, (b) or identified to a higher taxonomic resolution during bioinformatic analyses.

## Conclusion

We have demonstrated that landscape-level biodiversity for some common terrestrial and aquatic plant and animal groups can be estimated by sampling eDNA in rivers at the catchment scale. Our study shows that eDNA can be used to detect the unique spermatophyte assemblages of river catchments, even at the genus level. As the current biodiversity crisis grows, so does the need for cost-effective, repeatable and rapid biodiversity measurements at large spatial scales, such as using eDNA. Before implementing large-scale eDNA-based biomonitoring approaches, it is vital to understand how biological information is integrated within river networks, especially in highly diverse and spatially heterogeneous alpine habitats. Challenges remain in determining the most effective ways to capture the composition of plant assemblages using universal or other markers. Moreover, repeatable and rapid measures of biodiversity using eDNA require a deeper understanding of the temporal and seasonal dynamics. Armed with this knowledge, decision-makers and stakeholders can make more informed policy choices to maintain mountainous ecosystems, one of biodiversity's last refuges in the face of global change.

## Supplementary Information

Below is the link to the electronic supplementary material.

Supplementary file1 (DOCX 30 KB)

Supplementary file2 (XLSX 31 KB)

Supplementary file3 (DOCX 813 KB)

Supplementary file4 (XLSX 59 KB)

## Data Availability

All data available here: https://doi.org/10.16904/envidat.409.

## Code availability

Analysis code available here: https://doi.org/10.16904/envidat.409.
