# Supplementary material for: Catchment-based sampling of river eDNA integrates terrestrial and aquatic biodiversity of alpine landscapes
Source: Oecologia. 2023 Aug 9;202(4):699–713. doi: 10.1007/s00442-023-05428-4 (PMC10475001; doi:10.1007/s00442-023-05428-4)
Supplement: Supplementary file 1 — Supplementary file1 (DOCX 30 KB) [file 442_2023_5428_MOESM1_ESM.docx]

**Catchment-based sampling of river eDNA integrates terrestrial and aquatic biodiversity of alpine landscapes**

Merin Reji Chacko, Florian Altermatt, Fabian Fopp, Antoine Guisan, Thomas Keggin, Arnaud Lyet, Pierre-Louis Rey, Eilísh Richards, Alice Valentini, Conor Waldock, Loïc Pellissier

***Corresponding authors:** [**merin.rejichacko@gmail.com**](mailto:merin.rejichacko@gmail.com) **,** [**loic.pellissier@usys.ethz.ch**](mailto:loic.pellissier@usys.ethz.ch)

**Supplementary Information 1: Methods**

### Extraction

We performed two extractions per filter using the protocol of Pont et al. (2018). We agitated the filtration capsule containing the CL1 buffer for 15 min at 800 rpm using an S50 shaker (Ingenieurbuero^TM^) before emptying the buffer into two 50-ml tubes. The tubes were then centrifuged for 15 min at 15 000 g. After the supernatant was removed with a sterile pipette, each tube contained 15 ml of liquid. Next, we added 33 ml of ethanol and 1.5 ml of 3 M sodium acetate to each 50-ml tube. The tubes were stored at least for one night at -20 °C. Then the tubes were centrifuged again at 15 000 g for 15 min, at 6 °C and then supplemented with 720 μl of ATL buffer from the DNeasy Blood and Tissue Extraction Kit (Qiagen GmbH). Each tube was then vortexed before the supernatant was combined with 20 μl of Proteinase K in a 2-ml tube. The tubes were incubated for 2 hours at 56 °C. We used NucleoSpin (R) Soil (MACHEREY-NAGEL GmbH & Co.) to perform two DNA extractions per filter capsule, starting from step six and continuing according to the manufacturer's manual. 100 μl of SE buffer was used twice for the elution process. Negative extraction controls were processed in parallel.

### Amplification and Sequencing

Following established protocol (Biggs et al., 2015), samples were tested for inhibition; inhibited samples were diluted fivefold. 3 μl of DNA extract were used as a template to perform DNA amplification in a final volume of 25 μl. The amplification mixture consisted of 1 U of AmpliTaq Gold DNA Polymerase (Applied Biosystem), 10 mM Tris-HCl, 50 mM KCl, 2.5 mM MgCl2, 0.2 mM of each dNTP, 0.2 μm of each primer, and 0,2 μg/μl bovine serum albumin (BSA, Roche Diagnostic). eDNA was amplified using two primer sets targeting vertebrates (Vert01, forward: -TTAGATACCCCACTATGC, reverse: -TAGAACAGGCTCCTCTAG, mean marker length: 97 bp) and spermatophytes (g-h/Sper01, forward: -GGGCAATCCTGAGCCAA, reverse: CCATTGAGTCTCTl bGCACCTATC, mean marker length: 48 bp) (Taberlet et al., 2018). For the vertebrate primer, human blocking primer (De Barba et al., 2014) with a final concentration of 4 μM was also added to the mixture. The two primer sets were 5’-labelled with an eight-nucleotide tag unique to each sample (with at least three differences between any pair of tags), allowing each sequence to be assigned to the corresponding sample during sequence analysis. Identical tags were used for the forward and reverse primers. The PCR mixture was denatured at 95 °C for 10 min, followed by 50 cycles of 30 s at 95 °C, 30 s at 55 °C for Vert01 and 50 °C for Sper01 and 1 min at 72 °C, with a final elongation step at 72 °C for 7 min. We ran 12 PCR replicates per extracted DNA and per marker. Samples were first titrated using capillary electrophoresis (QIAxcel; Qiagen GmbH) and purified using the MinElute PCR purification kit, then titrated again. Purified PCR products were pooled in equal volumes, achieving a theoretical sequencing depth of 300 000 and 100 000 reads per sample for vertebrates and plants, respectively. Two libraries were prepared using the Metafast protocol (Fasteris). Two paired-end sequencing runs (2 × 125 bp) were carried out on a MiSeq sequencer (Illumina, San Diego, CA, USA) using the MiSeq Flow Cell Kit Version3 (Illumina, San Diego, CA, USA) according to the manufacturer's instructions. In order to monitor the possibility of contamination, four negative extraction controls and two negative PCR controls (12 replicates using ultrapure water) were amplified per primer pair and sequenced parallel to the samples.

### Bioinformatic analyses

Reads were processed post-sequencing in order to remove errors and then analysed using the OBITools package (Boyer et al., 2016) according to existing protocols (Polanco Fernández et al., 2021; Valentini et al., 2016). The ILLUMINAPAIREDEND program assembled forward and reverse reads, using a minimum score of 40 and retrieving only joined sequences. NGSFILTER software was used to assign each read to a sample. Using OBISPLIT, the original data was divided such that each sample had its own data set. Next, each sample was individually analysed, and then the taxon list was merged. For the final ecological analysis, OBIUNIQ clustered together strictly identical sequences. The OBIGREP program excluded sequences shorter than 20 bp, those with fewer than ten occurrences and those labelled "internal" by the OBICLEAN program. The ECOTAG program with the sequences extracted from release 142 of the ENA database (standard sequences) was then used for the remaining sequences' taxonomic assignment. Sequences with <100 reads in each sample were discarded, and only sequences with a similarity of higher than 98% for spermatophytes and 90% for vertebrates were kept. Taxonomic assignment outputs were corrected to avoid any over-confidence in assignments: species-level assignments were validated only for sequences with an identification match >98%, genus-level with a 96–98% match and family-level with a 90–96% match. Considering the incorrect assignment of a few sequences to the sample due to tag-jumps (Schnell et al., 2015), we discarded all sequences with a frequency of occurrence < 0.001 per sequence and per library. The extraction and PCR negative controls remained completely clean after the filtering pipeline, and the samples contained no sequence reads.

### Assessment of Total and Common Regional Species Richness

We calculated regional species richness as a sum of all species present in the ten catchments, and furthermore sorted species into common and rare classification to obtain the regional species richness of common species. For birds, we obtained regional species occurrence data from the Swiss Ornithological Institute Vogelwarte (<https://www.vogelwarte.ch/>), which provides species distribution maps validated from long term field expeditions. For all other vertebrates, we obtained regional species presence-absence data from InfoSpecies (Schweizerisches Informationszentrum für Arten, Swiss Information Centre for Species), which provides species occurrences validated from primary and secondary sources (academic research, monitoring programs, existing literature, collections, as well as field expeditions from environmental offices and cantonal efforts) at the 5 × 5 km scale. We next assigned species into common or rare categories using existing literature, expert opinion and records from InfoSpecies. For amphibians, the spatial data was first aggregated to the catchment level. Species were assigned as common if the species were present in at least half of the catchments according to the regional species occurrence data. The freely available spatial data only included presences and not the number of observations. Thus, to verify that species were not assigned as common due to a single observation in one catchment, an additional visual inspection of publicly available species distribution maps (<https://lepus.unine.ch/carto/>) with some information of the number of sightings was conducted. This confirmed that all species present within a catchment demonstrated multiple sightings across multiple grid cells within the catchment. Swiss breeding birds were divided into rare and common categories by a combination of both visual inspections of the territory maps from the Swiss Ornithological Institute and local expert knowledge of the area. Mammal and fish species were explored using species maps on the GBIF database (<https://www.gbif.org/>) and classified according to their percentage spatial coverage into very common (>75%), common (50–75%), fairly common (25–50%), uncommon (<25%) and rare (<10 locations). These classifications were aggregated into two classes: common (>25%) and rare (<25%). Moreover, two common exclusively lake species (*Salvelinus namaycush* and *Salvelinus umbla)* were reclassified as rare as they are not expected to be found in rivers. For spermatophytes, we obtained regional species occurrence data compiled from long-term field studies in the area (Guisan et al., 2014). Species were assigned as common if they were present in at least 5% of all sampled plots within the ten catchments. The regional species occurrence data did not include ornamental, agricultural, and lowland plants which are part of the national checklist. For this analysis, we excluded identified taxa that were not part of the regional species occurrence data so that taxa absent in the occurrence dataset would not be counted towards species accumulation rate. For the taxa identified at lower the species level resolution, we assigned them as rare if the taxon encompassed at least one species which was identified as rare, otherwise, it was classified as common.

### References

Biggs, J., Ewald, N., Valentini, A., Gaboriaud, C., Dejean, T., Griffiths, R. A., Foster, J., Wilkinson, J. W., Arnell, A., Brotherton, P., Williams, P., & Dunn, F. (2015). Using eDNA to develop a national citizen science-based monitoring programme for the great crested newt (Triturus cristatus). *Biological Conservation*, *183*, 19–28. https://doi.org/10.1016/j.biocon.2014.11.029

Boyer, F., Mercier, C., Bonin, A., Le Bras, Y., Taberlet, P., & Coissac, E. (2016). obitools: a unix-inspired software package for DNA metabarcoding. *Molecular Ecology Resources*, *16*(1), 176–182. https://doi.org/10.1111/1755-0998.12428

De Barba, M., Miquel, C., Boyer, F., Mercier, C., Rioux, D., Coissac, E., & Taberlet, P. (2014). DNA metabarcoding multiplexing and validation of data accuracy for diet assessment: Application to omnivorous diet. *Molecular Ecology Resources*, *14*(2), 306–323. https://doi.org/10.1111/1755-0998.12188

Guisan, A., Dubuis, A., & Vittoz, P. (2014). *Data from: Predicting spatial patterns of plant species richness: a comparison of direct macroecological and species stacking modelling approaches*. Dryad. https://doi.org/10.5061/dryad.28d4k

Polanco Fernández, A., Marques, V., Fopp, F., Juhel, J. B., Borrero-Pérez, G. H., Cheutin, M. C., Dejean, T., González Corredor, J. D., Acosta-Chaparro, A., Hocdé, R., Eme, D., Maire, E., Spescha, M., Valentini, A., Manel, S., Mouillot, D., Albouy, C., & Pellissier, L. (2021). Comparing environmental DNA metabarcoding and underwater visual census to monitor tropical reef fishes. *Environmental DNA*, *3*(1), 142–156. https://doi.org/10.1002/edn3.140

Pont, D., Rocle, M., Valentini, A., Civade, R., Jean, P., Maire, A., Roset, N., Schabuss, M., Zornig, H., & Dejean, T. (2018). Environmental DNA reveals quantitative patterns of fish biodiversity in large rivers despite its downstream transportation. *Scientific Reports*, *8*(1), 1–13. https://doi.org/10.1038/s41598-018-28424-8

Schnell, I. B., Sollmann, R., Calvignac-Spencer, S., Siddall, M. E., Yu, D. W., Wilting, A., & Gilbert, M. T. P. (2015). iDNA from terrestrial haematophagous leeches as a wildlife surveying and monitoring tool - prospects, pitfalls and avenues to be developed. *Frontiers in Zoology*, *12*(1), 1–14. https://doi.org/10.1186/s12983-015-0115-z

Taberlet, P., Bonin, A., Zinger, L., & Coissac, E. (2018). Environmental DNA: For biodiversity research and monitoring. In *Environmental DNA: For Biodiversity Research and Monitoring*. Oxford University Press. https://doi.org/10.1093/oso/9780198767220.001.0001

Valentini, A., Taberlet, P., Miaud, C., Civade, R., Herder, J., Thomsen, P. F., Bellemain, E., Besnard, A., Coissac, E., Boyer, F., Gaboriaud, C., Jean, P., Poulet, N., Roset, N., Copp, G. H., Geniez, P., Pont, D., Argillier, C., Baudoin, J. M., … Dejean, T. (2016). *Next-generation monitoring of aquatic biodiversity using environmental DNA metabarcoding*. *25*(4), 929–942. https://doi.org/10.1111/MEC.13428
