# Supplementary material for: Catchment-based sampling of river eDNA integrates terrestrial and aquatic biodiversity of alpine landscapes
Source: Oecologia. 2023 Aug 9;202(4):699–713. doi: 10.1007/s00442-023-05428-4 (PMC10475001; doi:10.1007/s00442-023-05428-4)

**Catchment-based sampling of river eDNA integrates terrestrial and aquatic biodiversity of alpine landscapes**

Merin Reji Chacko, Florian Altermatt, Fabian Fopp, Antoine Guisan, Thomas Keggin, Arnaud Lyet, Pierre-Louis Rey, Eilísh Richards, Alice Valentini, Conor Waldock, Loïc Pellissier

***Corresponding authors:** [**merin.rejichacko@gmail.com**](mailto:merin.rejichacko@gmail.com) **,** [**loic.pellissier@usys.ethz.ch**](mailto:loic.pellissier@usys.ethz.ch)

**Supplementary Information 3**

*Table S1.* **Arealstatistik land cover classifications.** The *Arealstatistik* land cover plant classification was modified into broad land cover types.

| **Arealstatistik Nomenklatur 2004 (English Translation)** | **Land Cover Types** |
| --- | --- |
| Industrial and commercial buildings | Urban |
| Conversion of industrial and commercial buildings | Urban |
| One and two-family houses | Urban |
| Conversion of single and two-family houses | Urban |
| Townhouses and terraced houses | Urban |
| Conversion of terraced and terraced houses | Urban |
| Apartment buildings | Urban |
| Conversion of multi-family houses | Urban |
| Public buildings | Urban |
| Relocation of public buildings | Urban |
| Agricultural buildings | Urban |
| Surroundings of agricultural buildings | Urban |
| Unspecified buildings | Urban |
| Relocation of unspecified buildings | Urban |
| Highways | Urban |
| Highway green | Urban |
| Roads, paths | Urban |
| Street green | Urban |
| Parking area | Urban |
| Fortified railway area | Urban |
| Track green | Urban |
| Airfields | Urban |
| Grass runways, airfield green | Urban |
| Power supply systems | Urban |
| Wastewater treatment plants | Urban |
| Other supply and disposal facilities | Urban |
| Landfills | Urban |
| Degradation | Urban |
| Construction sites | Urban |
| Construction and settlement wasteland | Urban |
| Public parks | Urban |
| Sports facilities | Urban |
| Golf courses | Urban |
| Campsites | Urban |
| Allotments | Urban |
| Graveyards | Urban |
| Orchards | Removed |
| Field fruit | Removed |
| Vineyards | Removed |
| Horticultural land | Removed |
| Farmland | Removed |
| Natural meadows | Low grassland |
| Home pastures | Low grassland |
| Bushy meadows and home pastures | Low grassland |
| Alpine meadows | High grassland |
| Favourable alpine and Jura pastures | High grassland |
| Bushy alpine and Jura pastures | High grassland |
| Petrified alpine and Jura pastures | High grassland |
| Sheep alps | High grassland |
| Normal forest | Forest |
| Narrow forest | Forest |
| Afforestations | Forest |
| Logging | Removed |
| Forest damage | Removed |
| Abandoned Forest (on agricultural land) | Forest |
| Cleared Forest (on unproductive land) | Forest |
| Scrub forest | Forest |
| Copses, hedges | Forest |
| Groups of trees (on agricultural land) | Removed |
| Groups of trees (on unproductive land) | Forest |
| Stagnant water | Water |
| Water courses | Water |
| Flood control | Urban |
| Shrubbery, shrubby vegetation | Shrubland |
| Unproductive grass and herb vegetation | Rock |
| Avalanche and rockfall barriers | Rock |
| Wetlands | Water |
| Alpine sports infrastructure | Urban |
| Rock | Rock |
| Boulders, sand | Rock |
| Landscape interventions | Urban |
| Glaciers, firns | Rock |

*Table S2.* **Plant habitat classifications.** The *Flora Indicativa* plant classification was used. The *Flora Indicativa* broader classification groups were reassigned into broad land cover types.

| **ID** | **Flora Indicativa Classification** | **Flora Indicativa Broad Classification** | **Land Cover Types** |
| --- | --- | --- | --- |
| 1.1 | Dormant waters, lakes, small lakes | Water bodies | Water |
| 1.2 | Flowing waters | Water bodies | Water |
| 1.3 | Aquatic habitats (banks, ponds, ditches, oxbow lakes, reed beds, large sedge swamps, bog pools, ravines | Water bodies | Water |
| 1.4 | Flooded habitats, at times with strongly fluctuating water balance (troughs, ditches, banks, cart tracks, wallows) | Water bodies | Water |
| 2.1 | Fields, crops, vineyards, olive groves, fallow land | Nitrophilic land vegetation, natural or influenced by humans | Removed |
| 2.2 | Ruderal and half ruderal corridors (paths, debris, landfills, dung heaps, surroundings of walls and buildings, railway systems, embankments, step corridors, pavement) | Nitrophilic land vegetation, natural or influenced by humans | Removed |
| 2.3 | Hedges, forest edges, clearings | Nitrophilic land vegetation, natural or influenced by humans | Removed |
| 2.4 | Cattle store, balms | Nitrophilic land vegetation, natural or influenced by humans | Removed |
| 2.5 | Embankments, fringes of watercourses, alluvion gravel banks | Nitrophilic land vegetation, natural or influenced by humans | Removed |
| 2.6 | Forest cuts, clearings, forest roads, burns | Nitrophilic land vegetation, natural or influenced by humans | Removed |
| 2.7 | Parks, lawns, gardens, avenues, cemeteries, sports fields | Nitrophilic land vegetation, natural or influenced by humans | Removed |
| 2.8 | Fruit and chestnut plantations (high and low trunk crops) | Nitrophilic land vegetation, natural or influenced by humans | Removed |
| 2.9 | Cultivated plants | Nitrophilic land vegetation, natural or influenced by humans | Removed |
| 3.1 | Sandy rock outcrops | Rock outcrops, sand, tuff, rocks, scree, gravel pits, stone quarries | Rock |
| 3.2 | Rocks and walls (rock faces, walls, balms, caves, rock foot) | Rock outcrops, sand, tuff, rocks, scree, gravel pits, stone quarries | Removed |
| 3.3 | Scree in the broader sense (rubble corridors, moraine, landslides, block fields, ruins) | Rock outcrops, sand, tuff, rocks, scree, gravel pits, stone quarries | Rock |
| 3.4 | Karst fields, karst corridors | Rock outcrops, sand, tuff, rocks, scree, gravel pits, stone quarries | Rock |
| 3.5 | Gravel pits, stone quarries, marl pits | Rock outcrops, sand, tuff, rocks, scree, gravel pits, stone quarries | Removed |
| 4.1 | Springs, spring corridors, rivulets, tuffs, waterfalls, cascades | Springs, streams | Water |
| 4.2 | Brook edges | Springs, streams | Water |
| 5.1 | Flat moors and transition moors | Moors | Water |
| 5.2 | Raised moors | Moors | Water |
| 6.1 | Mediterranean dry grasses (annual grasses) | Meadows, pastures, lawns, snow pelts, herbaceous forests | Low grassland |
| 6.2 | Dry and semi-arid lawns, meadows and pastures from foothills to subalpine level, steppe lawns, rocky lawns, stone corridors | Meadows, pastures, lawns, snow pelts, herbaceous forests | Low grassland |
| 6.3 | Meso- to eutrophic hygrophilic meadows and pastures | Meadows, pastures, lawns, snow pelts, herbaceous forests | Low grassland |
| 6.4 | Moist acidophilic rough meadows, pastures and lawns | Meadows, pastures, lawns, snow pelts, herbaceous forests | Low grassland |
| 6.5 | Subalpine-alpine lawns in the broader sense, including rocky lawns | Meadows, pastures, lawns, snow pelts, herbaceous forests | High grassland |
| 6.6 | Snow valleys | Meadows, pastures, lawns, snow pelts, herbaceous forests | High grassland |
| 6.7 | Thermophilic forest edges | Meadows, pastures, lawns, snow pelts, herbaceous forests | Low grassland |
| 7.1 | Dwarf shrub heather in the broader sense | Dwarf shrub heaths, tall herbaceous vegetation, garrigue, shrubs in the broader sense | Shrub |
| 7.2 | Tall herbaceous corridors, nitrophilic veil societies, fern corridors | Dwarf shrub heaths, tall herbaceous vegetation, garrigue, shrubs in the broader sense | Shrub |
| 7.3 | Low garrigue, low maquis | Dwarf shrub heaths, tall herbaceous vegetation, garrigue, shrubs in the broader sense | Shrub |
| 7.4 | Shrubs, hedges, forest edges, brushwood, pioneer forests | Dwarf shrub heaths, tall herbaceous vegetation, garrigue, shrubs in the broader sense | Shrub |
| 7.5 | Low riverside willow shrubs | Dwarf shrub heaths, tall herbaceous vegetation, garrigue, shrubs in the broader sense | Water |
| 7.6 | Tall Mediterranean shrubs | Dwarf shrub heaths, tall herbaceous vegetation, garrigue, shrubs in the broader sense | Shrub |
| 8.1 | Green alder shrubs, subalpine willow shrubs | Shrubland, in the broader sense | Forest |
| 8.2 | *Pinus mugo* shrubs | Shrubland, in the broader sense | Forest |
| 9.1 | Coniferous forests | Forests | Forest |
| 9.2 | Mesophilic deciduous forests | Forests | Forest |
| 9.3 | Sub-Mediterranean thermophilic oak and hop beech forests | Forests | Forest |
| 9.4 | Mediterranean evergreen oak forests | Forests | Forest |
| 9.5 | *Robinia* plantations and secondary forests with *Robinia* | Forests | Removed |

*Table S3* **Monte Carlo permutation test results for dbRDA on land cover proportions at 250, 500 and 1 000 m radii buffers, and elevation (vertebrates).** We assessed the marginal effects of the absolute elevation, as well as fractions of land cover (forest, high grassland, low grassland, urban, shrub, water, rock) to the 30 vertebrate assemblages by way of the Jaccard dissimilarity index. For the 1 000 m analysis, high grassland, rock and water proportion were removed due to high correlation.

| **Buffer radius (m)** | **Constraint** | **Df** | **SumOfSqs** | **F** | **Pr(>F)** |
| --- | --- | --- | --- | --- | --- |
| 250 | Forest | 1 | 0.245 | 0.74 | 0.803 |
|  | High grassland | 1 | 0.309 | 0.934 | 0.546 |
|  | Low grassland | 1 | 0.407 | 1.232 | 0.181 |
|  | Shrubland | 1 | 0.279 | 0.843 | 0.738 |
|  | Urban | 1 | 0.25 | 0.757 | 0.784 |
|  | Water | 1 | 0.359 | 1.085 | 0.335 |
|  | Rock | 1 | 0.247 | 0.747 | 0.844 |
|  | Absolute Elevation | 1 | 0.356 | 1.075 | 0.381 |
|  | Residual | 21 | 6.945 |  |  |
| 500 | Forest | 1 | 0.225 | 0.68 | 0.905 |
|  | High grassland | 1 | 0.251 | 0.758 | 0.83 |
|  | Low grassland | 1 | 0.283 | 0.854 | 0.711 |
|  | Shrubland | 1 | 0.264 | 0.796 | 0.784 |
|  | Urban | 1 | 0.233 | 0.703 | 0.863 |
|  | Water | 1 | 0.343 | 1.036 | 0.416 |
|  | Rock | 1 | 0.271 | 0.818 | 0.774 |
|  | Absolute Elevation | 1 | 0.314 | 0.948 | 0.562 |
|  | Residual | 21 | 6.951 |  |  |
| 1 000 | Forest | 1 | 0.371 | 1.152 | 0.251 |
|  | Low grassland | 1 | 0.299 | 0.927 | 0.561 |
|  | Shrubland | 1 | 0.376 | 1.168 | 0.212 |
|  | Urban | 1 | 0.305 | 0.948 | 0.574 |
|  | Absolute Elevation | 1 | 0.418 | 1.296 | 0.112 |
|  | Residual | 24 | 7.731 |  |  |

*Table S4* **Monte Carlo permutation test results for dbRDA on land cover proportions at 250, 500 and 1 000 m radii buffers, and elevation (spermatophytes).** We assessed the marginal effects of the absolute elevation, as well as fractions of land cover (forest, high grassland, low grassland, urban, shrub, water, rock) to the 30 spermatophyte assemblages by way of the Jaccard dissimilarity index. For the 1 000 m analysis, high grassland, rock and water proportion were removed due to high correlation.

| **Buffer radius (m)** | **Constraint** | **Df** | **SumOfSqs** | **F** | **Pr(>F)** |
| --- | --- | --- | --- | --- | --- |
| 250 | Forest | 1 | 0.162 | 1.067 | 0.362 |
|  | High grassland | 1 | 0.147 | 0.97 | 0.479 |
|  | Low grassland | 1 | 0.193 | 1.274 | 0.166 |
|  | Shrubland | 1 | 0.148 | 0.977 | 0.444 |
|  | Urban | 1 | 0.12 | 0.789 | 0.704 |
|  | Water | 1 | 0.283 | 1.867 | 0.025 |
|  | Rock | 1 | 0.191 | 1.257 | 0.214 |
|  | Absolute Elevation | 1 | 0.272 | 1.796 | 0.032 |
|  | Residual | 21 | 3.184 |  |  |
| 500 | Forest | 1 | 0.173 | 1.129 | 0.308 |
|  | High grassland | 1 | 0.185 | 1.207 | 0.252 |
|  | Low grassland | 1 | 0.146 | 0.95 | 0.482 |
|  | Shrubland | 1 | 0.209 | 1.361 | 0.153 |
|  | Urban | 1 | 0.135 | 0.877 | 0.616 |
|  | Water | 1 | 0.197 | 1.282 | 0.173 |
|  | Rock | 1 | 0.155 | 1.007 | 0.414 |
|  | Absolute Elevation | 1 | 0.235 | 1.529 | 0.075 |
|  | Residual | 21 | 3.222 |  |  |
| 1 000 | Forest | 1 | 0.167 | 1.03 | 0.364 |
|  | Low grassland | 1 | 0.103 | 0.638 | 0.907 |
|  | Shrubland | 1 | 0.186 | 1.144 | 0.303 |
|  | Urban | 1 | 0.098 | 0.604 | 0.941 |
|  | Absolute Elevation | 1 | 0.404 | 2.493 | 0.001 |
|  | Residual | 24 | 3.892 |  |  |

*Table S5* **Kendall's Tau-B statistic for the comparison of catchment-level land cover type proportions.** We compared the relationship between *Flora Indicativa* assignments of plant genera to remotely sensed land cover fractions across entire catchments. The urban fraction was correlated with the fraction of non-native plants.

| Land Cover Type | COR | p-value |
| --- | --- | --- |
| High grassland | 0.176 | 0.501 |
| Low grassland | -0.378 | 0.155 |
| Rock | 0.111 | 0.727 |
| Shrub | 0.225 | 0.369 |
| Urban | 0.024 | 0.926 |
| Water | 0.333 | 0.216 |
| Forest | 0.289 | 0.291 |

*Table S6* **Kendall's Tau-B statistic for the comparison of site-level land cover type proportions.** We compared the relationship between *Flora Indicativa* assignments of plant genera to remotely sense land cover fractions at 250 m, 500 m and 1000 m buffers around each sampling site. The urban fraction was correlated with the fraction of non-native plants.

| **Buffer radius (m)** | **Land Cover Type** | **COR** | **p-value** |
| --- | --- | --- | --- |
| 250 | Forest | 0.068 | 0.638 |
|  | High grassland | 0.403 | 0.011 |
|  | Low grassland | -0.114 | 0.450 |
|  | Rock | -0.275 | 0.099 |
|  | Shrub | -0.132 | 0.425 |
|  | Urban | 0.086 | 0.579 |
|  | Water | -0.163 | 0.326 |
| 500 | Forest | 0.086 | 0.549 |
|  | High grassland | 0.314 | 0.043 |
|  | Low grassland | -0.150 | 0.308 |
|  | Rock | -0.042 | 0.790 |
|  | Shrub | -0.067 | 0.680 |
|  | Urban | 0.239 | 0.113 |
|  | Water | -0.173 | 0.265 |
| 1000 | Forest | 0.077 | 0.593 |
|  | High grassland | 0.263 | 0.084 |
|  | Low grassland | -0.229 | 0.116 |
|  | Rock | 0.009 | 0.948 |
|  | Shrub | 0.013 | 0.929 |
|  | Urban | 0.283 | 0.059 |
|  | Water | -0.142 | 0.332 |

*Figure S1.* **Species accumulation curves across filter replicates for amphibians, birds, fishes, mammals, and seed-bearing plants including potentially redundant taxa.** The darker blue line represents total regional species richness, and the lighter blue line represents common regional species richness. The solid curves represent multimodel mean averages for the 30 replicates sampled, while the dashed curves represent extrapolated values for a further 20 replicates. The darker shades represent all found taxa, while the lighter shades represent common taxa.


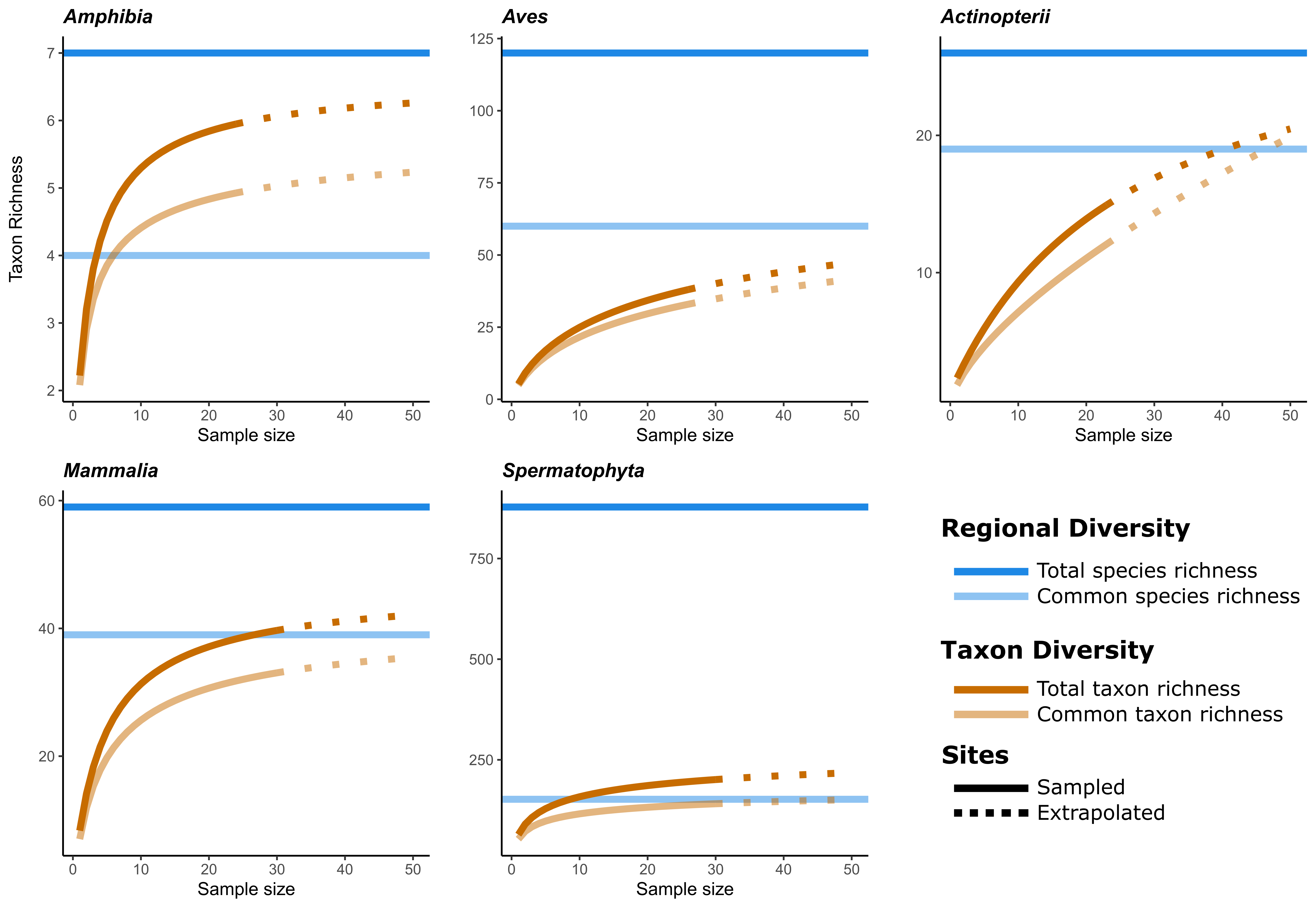

Supplement: Supplementary file 3 — Supplementary file3 (DOCX 813 KB) [file 442_2023_5428_MOESM3_ESM.docx]
